# Human Cytomegalovirus-Infected Glioblastoma Cells Display Stem Cell-Like Phenotypes

**DOI:** 10.1128/mSphere.00137-17

**Published:** 2017-06-21

**Authors:** Che Liu, Paul A. Clark, John S. Kuo, Robert F. Kalejta

**Affiliations:** a Institute for Molecular Virology and McArdle Laboratory for Cancer Research, University of Wisconsin—Madison, Madison, Wisconsin, USA; b Department of Neurological Surgery, University of Wisconsin—Madison, Madison, Wisconsin, USA; University of Arizona

**Keywords:** brain cancer, cancer, chemoresistance, chemotherapy, herpesvirus

## Abstract

Glioblastoma multiforme (GBM) is the most common brain tumor in adults. Human cytomegalovirus (HCMV) genomes are present in GBM tumors, yielding hope that antiviral treatments could prove therapeutic and improve the poor prognosis of GBM patients. We discovered that GBM cells infected *in vitro* with HCMV display properties of cancer stem cells. HCMV-infected GBM cells grow more slowly than mock-infected controls, demonstrate a higher capacity for self-renewal determined by a sphere formation assay, and display resistance to the chemotherapeutic drug temozolomide. Our data suggest that HCMV, while present in only a minority of the cells within a tumor, could contribute to the pathogenesis of GBMs by promoting or prolonging stem cell-like phenotypes, thereby perpetuating tumors in the face of chemotherapy. Importantly, we show that temozolomide sensitivity is restored by the antiviral drug ganciclovir, indicating a potential mechanism underlying the positive effects observed in GBM patients treated with antiviral therapy.

**IMPORTANCE** A role for HCMV in GBMs remains controversial for several reasons. Some studies find HCMV in GBM tumors, while others do not. Few cells within a GBM may harbor HCMV, making it unclear how the virus could be contributing to the tumor phenotype without infecting every cell. Finally, HCMV does not overtly transform cells *in vitro*. However, tumors induced by other viruses can be treated with antiviral remedies, and initial results indicate that this may be true for anti-HCMV therapies and GBMs. With such a poor prognosis for GBM patients, any potential new intervention deserves exploration. Our work here describes an evidence-based model for how HCMV could contribute to GBM biology while infecting very few cells and without transforming them. It also illuminates why anti-HCMV treatments may be beneficial to GBM patients. Our observations provide blueprints for future *in vitro* studies examining how HCMV manipulates stem cell-specific pathways and future clinical studies of anti-HCMV measures as GBM therapeutics.

## Abstract (precis)

A role for HCMV in GBMs remains controversial for several reasons. Some studies find HCMV in GBM tumors, while others do not. Few cells within a GBM may harbor HCMV, making it unclear how the virus could be contributing to the tumor phenotype without infecting every cell. Finally, HCMV does not overtly transform cells *in vitro*. However, tumors induced by other viruses can be treated with antiviral remedies, and initial results indicate that this may be true for anti-HCMV therapies and GBMs. With such a poor prognosis for GBM patients, any potential new intervention deserves exploration. Our work here describes an evidence-based model for how HCMV could contribute to GBM biology while infecting very few cells and without transforming them. It also illuminates why anti-HCMV treatments may be beneficial to GBM patients. Our observations provide blueprints for future *in vitro* studies examining how HCMV manipulates stem cell-specific pathways and future clinical studies of anti-HCMV measures as GBM therapeutics.

## INTRODUCTION

Glioblastoma multiforme (GBM) is the most aggressive and most common primary glioma in adults (1). Approximately 50% of adult gliomas and 10% of pediatric gliomas are GBMs. Surgical resection, radiation, and chemotherapy with temozolomide are the standard of care. These conventional treatments have modest efficacy. The median survival of GBM patients is less than 15 months (2), and the 5-year survival is ~5% (1). Novel therapies are desperately needed.

Viruses cause ~15% of all human cancers (3). In most cases, transforming viral oncoproteins are expressed in all cells within a tumor, and their expression is required for tumor maintenance. Therefore, targeting viral functions is a *bona fide* therapeutic option for these tumors. Indeed, prophylactic vaccines targeting cancer-causing viruses have (hepatitis B) (4) or likely will (human papillomavirus) (5) decrease virus-induced cancer incidence, and chemotherapeutic regimens have (human immunodeficiency virus) (6) or likely will (hepatitis C virus) (7) decrease virus-induced cancer incidence. Therefore, identifying other cancers with etiologies or courses driven by viral infection increases available therapeutic options.

Human cytomegalovirus (HCMV) is a betaherpesvirus (8) that encodes a variety of proteins that when expressed individually, or combined during infection, elicit all of the defined hallmarks of human cancers (9, 10). HCMV DNA genomes and protein antigens have been detected in GBM tumors (11–13). Preliminary clinical studies with chemical (14, 15) or immunological (16, 17) interventions against HCMV have proven effective at improving GBM patient outcome. However, not all examinations of GBM samples have detected viral genomes or antigens (18). A possible explanation for why some studies failed to detect HCMV in GBMs comes from our own work. We identified HCMV genomes present in the majority of GBM specimens examined, but we also determined that only a small minority of the cells within those tumors could be infected with the virus (19). While the low level of virus present in tumors seems to clarify why not all studies detect HCMV in GBMs (different studies have different detection limits), it also raises the issue of how HCMV might be affecting tumor biology while present in only a minority of tumor cells.

In order to determine whether infection of only a minority of cells could confer a growth or survival advantage to a GBM tumor, we examined HCMV-positive primary GBM tumor cells cultured *ex vivo*. We discovered that HCMV-infected GBM cells display properties *in vitro* similar to those of cancer stem cells. Furthermore, we suggest that the ability of HCMV to engender such a phenotype may promote tumor recurrence after treatment and may explain the promising initial results of chemotherapeutic and immunologic anti-HCMV regimens for GBM patients.

## RESULTS

### Viral genomes are not detected after *ex vivo* culture of HCMV-positive GBM tumors.

**FIG 1:**
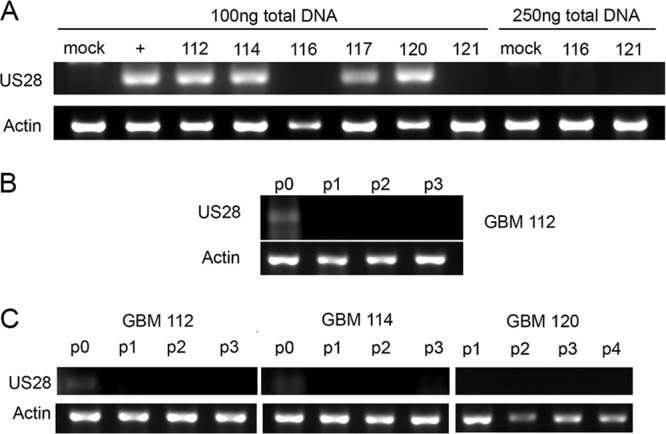
Viral genomes are not detected after *ex vivo* culture of HCMV-positive GBM tumors. (A) DNA isolated from snap-frozen GBM tumors was used as the template for PCR amplification of the HCMV Us28 gene or cellular actin. PCR products were analyzed by agarose gel electrophoresis with ethidium bromide staining. HCMV TB40/E-infected fibroblasts were used as a positive control (+). Mock-infected fibroblasts were used as a negative control. (B) GBM 112 cells were grown in monolayer culture for the indicated number of passages (passage 0 [p0] to passage 3 [p3]) and analyzed as described above. (C) GBM 112, GBM 114, and GBM 120 cells were grown as spheres for the indicated number of passages and analyzed as described above. Images are representative of triplicate PCR experiments.

Four out of six snap-frozen GBM surgery samples tested positive for the presence of HCMV DNA (Fig. 1A). Increasing assay sensitivity by analyzing 2.5-fold-more input template failed to identify HCMV genomes in the negative samples (Fig. 1A). We conclude that our GBM 112, GBM 114, GBM 117, and GBM 120 cell samples are infected with HCMV, and GBM 116 and GBM 121 cell samples are not. We plated HCMV-positive GBM 112 cells as monolayers in serum-containing media (passage zero [P0]) and split the cultures three times (passage 1 [P1], P2, and P3). HCMV DNA was found in the P0 cells but was not detected after passage (Fig. 1B). We also cultured HCMV-positive GBM 112, GBM 114, and GBM 120 under sphere growth conditions in defined medium (20). HCMV DNA was found in the P0 cultures of GBM 112 and 114 but was not detected after passage (Fig. 1C). We conclude that viral genomes rapidly become undetectable after *ex vivo* culture of HCMV-positive GBM tumors.

### Primary GBM cells are permissive to HCMV *in vitro*.

**FIG 2:**
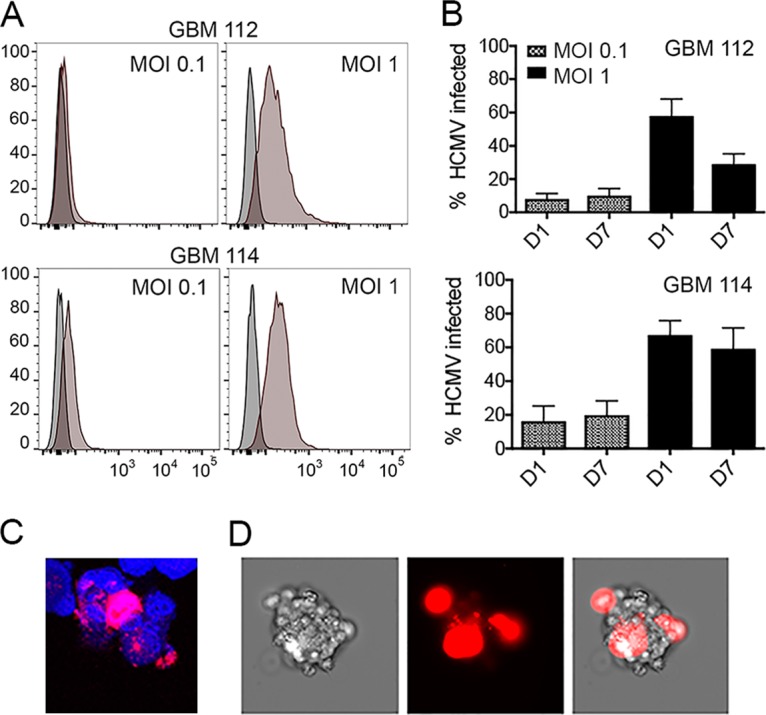
Primary GBM cells are permissive to HCMV *in vitro*. (A) GBM 112 and GBM 114 cells were infected with HCMV TB40/E-mCherry at the indicated MOI for 24 h and then analyzed by flow cytometry for mCherry expression (brown histograms). Mock-infected cells (gray histogram) served as a control. The image is representative of three biological replicates. (B) The average percentage of mCherry-positive (HCMV-infected) cells at 1 day (D1) or 7 days (D7) postinfection for three biological replicates of the experiment presented in panel A are presented. Error bars represent the standard errors of the means for the three biological replicates. (C) GBM 112 spheres infected for 5 days at a MOI of 1.0 with HCMV TB40/E-mCherry (red) and counterstained with 4′,6′-diamidino-2-phenylindole (DAPI) (blue) were imaged by confocal fluorescence microscopy. (D) GBM 112 spheres infected for 7 days at a MOI of 1.0 with HCMV TB40/E-mCherry (red) were imaged by conventional fluorescence microscopy. (Left) Bright-field image; (center) dark-field fluorescence image; (right) bright-field fluorescence image.

Because the natural *in vivo* HCMV infection was not efficiently maintained *in vitro*, we tested our cultured, primary GBM cells for susceptibility to infection *in vitro* with recombinant HCMV. For these experiments, we cultured GBM cells as spheres because this method better approximates the growth conditions of the parental tumor (21). GBM 112 and GBM 114 were infected at a low (0.1) or high (1.0) multiplicity of infection (MOI) with a recombinant clinical strain of HCMV expressing the fluorescent mCherry protein from the simian virus 40 (SV40) promoter. All infections generated mCherry-positive cells (Fig. 2A). At a MOI of 1, 58% ± 10.2% of GBM 112 cells and 63.9% ± 10.9% of GBM 114 cells were mCherry positive, while 8.1% ± 3.3% of GBM 112 cells and 16.2% ± 9.2% of GBM 114 cells were mCherry positive at a MOI of 0.1 (Fig. 2B). mCherry-positive cells were still discernible after 7 days (Fig. 2B). Confocal fluorescence microscopy (Fig. 2C) and bright-field conventional fluorescence microscopy (Fig. 2D) revealed the spatial orientation of infected cells within spheres.

**FIG 3:**
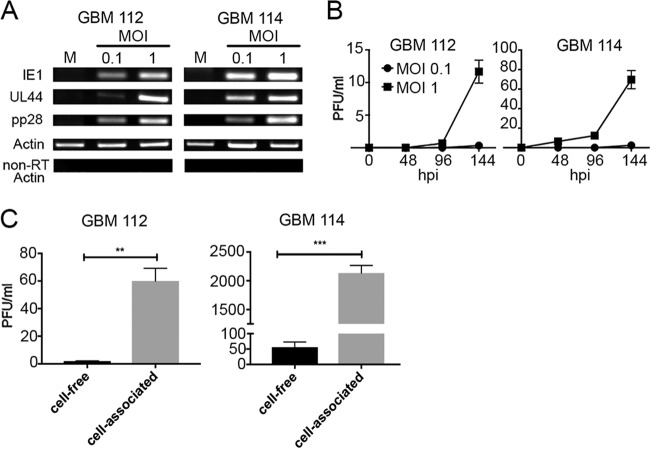
Primary GBM cells support productive HCMV replication *in vitro*. (A) RNA isolated from GBM 112 and GBM 114 cells infected at the indicated MOI with HCMV TB40/E-mCherry for 72 h was analyzed by RT-PCR for the indicated genes. Mock-infected cells (M) were used as the control. The image is representative of three biological replicates. (B) Supernatants collected at the indicated time (in hours postinfection [hpi]) from GBM 112 and GBM 114 cells infected with HCMV TB40/E-mCherry at the indicated MOI were subjected to a standard plaque assay. Duplicate biological assays were performed. (C) Progeny virus from cell-free or cell-associated samples of GBM 112 or GBM 114 cells infected at a MOI of 1.0 for 144 h were quantitated by a plaque assay. Thee biological replicate experiments were performed and analyzed by Student’s *t* test for statistical significance. **, *P* < 0.01; ***, *P* < 0.001.

Prior viral immediate early (IE) gene expression is required for activation of the SV40 promoter when incorporated into recombinant HCMV (22). Therefore, we hypothesized that HCMV initiates productive (lytic) phase viral gene expression when it infects *in vitro*-cultured GBM cells. Indeed, we detected transcripts from infected GBM cells (Fig. 3A) representing all kinetic classes of viral genes, including immediate early (IE1), early (UL44), and late (pp28). Both GBM 112 and GBM 114 cells released low levels of infectious progeny virions after high-MOI infection (Fig. 3B). Significantly higher levels of cell-associated progeny virus were quantitated (Fig. 3C). We conclude that HCMV productively infects primary GBM cells grown as spheres *in vitro*.

### GBM cells infected *in vitro* with HCMV divide less often than uninfected cells do.

**FIG 4:**
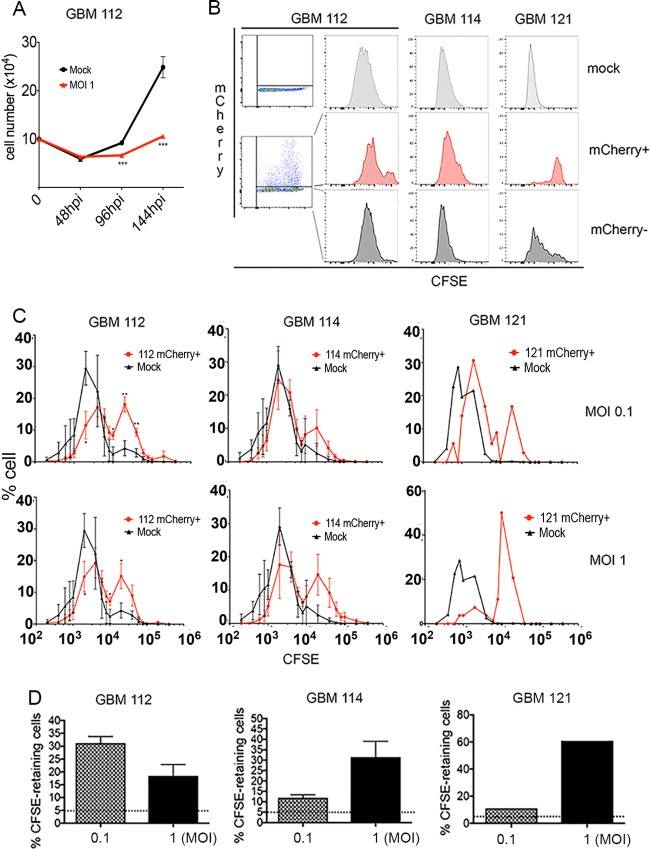
GBM cells infected *in vitro* with HCMV divide less often than uninfected cells do. (A) GBM 112 cells mock infected or infected at the indicated MOI with HCMV TB40/E-mCherry were grown as spheres, and viable cells were counted at the indicated time (in hours postinfection [hpi]). Averages of three biological replicates are plotted. Values that are significantly different (*P* < 0.001 by Student’s *t* test) are indicated (***). (B) Mock-infected or HCMV TB40/E-mCherry-infected GBM cells were loaded with CFSE and then cultured as spheres for 7 days at which time mCherry and CFSE fluorescence of viable cells were quantitated by flow cytometry. For infected cells, the mCherry-negative (mCherry-) and mCherry-positive (mCherry+) populations are shown. The graphs for GBM 112 and GBM 114 cells are representative of three biological replicates. (C) The number of cells within bins representing 1/5th log_10_ windows is plotted versus the upper limit of the CFSE window for three biological replicates of GBM 112 and GBM 114 cells treated as described above for panel B and one biological replicate of GBM 121 cells. Values that are significantly different by Student’s *t* test are indicated by asterisks as follows: **, *P* < 0.01; *, *P* < 0.05. (D) Cells within the gate representing the top 5% of CFSE staining and mock-infected cells (not shown) were quantified for GBM 112, GBM 114, and GBM 121 cells infected with HCMV at the indicated MOI for 7 days. Averages are plotted with error bars representing the standard errors of the means for biological triplicate samples where appropriate.

Oncogenic proteins from oncogenic viruses inactivate cellular tumor suppressors and stimulate cell division (3, 9). HCMV encodes multiple proteins with these same abilities (23). However, we found that HCMV-infected GBM 112 cells divided at a rate that was lower than the rate of their uninfected counterparts (Fig. 4A). To better quantify cell divisions, we loaded cells with the fluorescent dye carboxyfluorescein succinimidyl ester (CFSE) and then monitored signal dilution as a marker for cell division. We used cells mock infected for 7 days to set the baseline levels for mCherry and CFSE fluorescence. HCMV-infected (mCherry-positive) cells showed higher CFSE fluorescence than mock-infected cells or the mCherry-negative (uninfected) population of cells within the cultures inoculated with virus (Fig. 4B), indicating that virally infected cells divided less frequently than uninfected cells do. This slow growth phenotype was observed (Fig. 4B) for both previously HCMV-positive tumors (GBM 112 and GBM 114) analyzed in three biological replicate experiments as well as a tumor (GBM 121) not naturally infected (Fig. 1A) and analyzed in a single experiment. To quantitate and, where applicable, statistically analyze this effect, we binned cells in 1/5th log_10_ windows and plotted the percentage of cells within that window versus the CFSE intensity of those cells (Fig. 4C). HCMV-infected GBM 112 cells with high CFSE levels were statistically overrepresented compared to mock-infected cells. HCMV-infected GBM 114 cells were also overrepresented in cells with high CFSE levels, although this difference was not statistically significant likely due to the range of the data. HCMV-infected GBM 121 cells could not be analyzed statistically but clearly showed more high-level CFSE cells than mock-infected cultures did. A more conventional analysis (24) is to define the slow-cycling population as the cells within the mock-infected group with the highest 5% of CFSE intensity and then quantitate the number of experimentally treated cells with equal or higher CFSE levels. With this test, we found that HCMV infection increased the number of slow-cycling cells within the population for all MOIs and GBM tumors analyzed (Fig. 4D). We conclude that populations of GBM cells infected *in vitro* with HCMV display a higher percentage of slow-growing cells than uninfected cell populations do.

### GBM cells infected *in vitro* with HCMV show increased sphere-forming ability.

**FIG 5:**
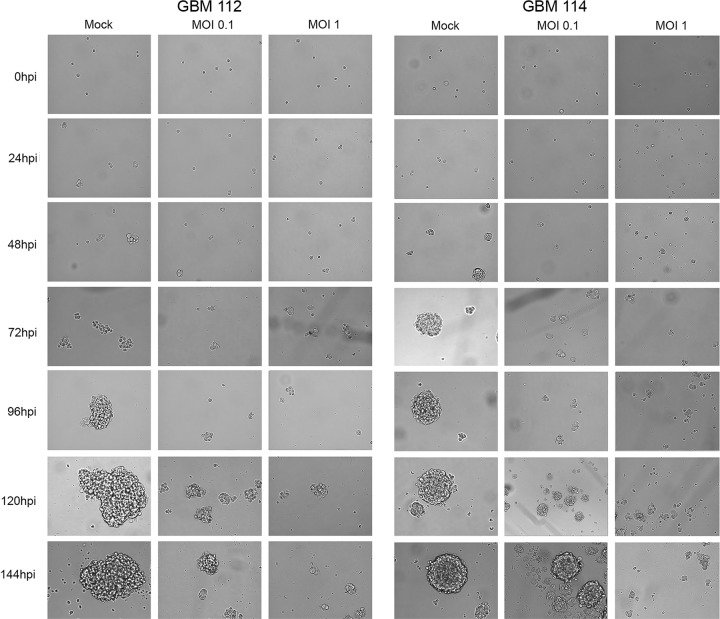
GBM cells infected *in vitro* with HCMV grow as spheres. GBM 112 and GBM 114 cells infected with HCMV TB40/E-mCherry at the indicated MOI were grown as spheres. At the indicated times, images representing randomly selected (presumably different) spheres were captured. Mock-infected cells served as a control.

**FIG 6:**
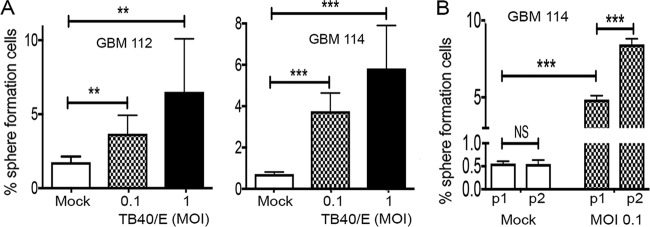
GBM cells infected *in vitro* with HCMV show increased sphere-forming ability. (A) GBM 112 and GBM 114 cells infected with HCMV TB40/E-mCherry at the indicated MOI were subjected to a sphere formation assay as described in Materials and Methods. Mock-infected cells were used as the control. The average percentages of sphere-forming cells from three biological replicates are plotted with error bars representing the standard errors of the means. ***, *P* < 0.001; **, *P* < 0.01. (B) A serial sphere assay (passage 1 [P1] and passage 2 [P2]) was conducted with GBM 114 cells infected with HCMV TB40/E-mCherry at the indicated MOI, analyzed, and displayed as in panel A. Serially passaged mock-infected cells were used as the control. ***, *P* < 0.001; NS, not significant by Student’s *t* test.

Such a CFSE retention assay is often used to identify the cancer stem cell component of a mixed population based upon their slow growth (25–27). Our data demonstrating that HCMV promoted a slow-growth, stem cell-like phenotype in GBM cells led us to ask whether HCMV-infected GBM cells displayed another property of GBM stem cells, namely, the ability to form spheres. GBM spheres grown in cell culture initiate from GBM stem cells, but the majority of the daughter cells within the growing sphere differentiate and lose the ability to rederive a new sphere (28). In our sphere growth assays (Fig. 5), 1.8% of GBM 112 cells and 0.71% of GBM 114 cells showed sphere-forming ability (Fig. 6A). However, HCMV-infected GBM 112 or GBM 114 cultures showed a much higher propensity to generate spheres upon dissociation (Fig. 6A, 6.5% for GBM 112 and 5.8% for GBM 114 cells infected at a MOI of 1), indicating the presence of a higher number of cells with this stem cell-like self-renewal property. For cells infected at a low MOI, this phenotype extended to the second generation, where the frequency of cells that displayed sphere-forming potential increased 1.7-fold compared to the previous passage (Fig. 6B). We conclude that HCMV-infected GBM cells show enhanced sphere-forming potential and an increased capacity for self-renewal.

### GBM cells infected *in vitro* with HCMV resist growth inhibition by temozolomide.

**FIG 7:**
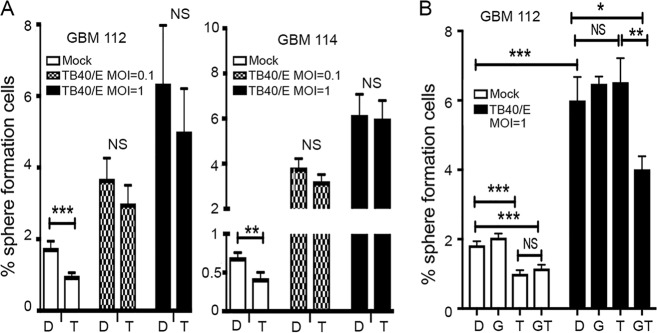
GBM 112 and GBM 144 cells infected *in vitro* with HCMV resist growth inhibition by temozolomide. (A) Sphere formation assays were conducted as described in the legend to Fig. 6 in the presence of carrier (DMSO [D]) or temozolomide (T) where indicated. The average percentages of sphere-forming cells from three biological replicates of  GBM 112 and GBM 144 cells (mock infected or infected with HCMV TB40/E) are plotted with error bars representing the standard errors of the means. ***, *P* < 0.001; **, *P* < 0.01; NS, not significant by Student’s *t* test. (B) Sphere formation assays were conducted as described above with the addition of ganciclovir (G) where indicated. The average percentages of sphere-forming cells from three biological replicates from GBM 112 cells (mock infected or infected with HCMV TB40/E) are plotted with error bars representing the standard errors of the means. ***, *P* < 0.001; **, *P* < 0.01; *, *P* < 0.05; NS, not significant by Student’s *t* test.

In addition to the slow growth and capacity for self-renewal shared by cancer stem cells and HCMV-infected GBMs, cancer stem cells also display therapeutic resistance (29, 30). Temozolomide is the current standard oral DNA alkylating chemotherapy for GBM patients (31). Therefore, we asked whether HCMV-infected GBM cells were resistant to temozolomide *in vitro*. We selected doses of temozolomide that inhibited the sphere-forming ability of mock-infected GBM cells by ~50% (Fig. 7A). We found that HCMV-infected GBM 112 and GBM 114 cultures formed spheres in the presence of temozolomide as well as they did in its absence (Fig. 7A). Treating infected GBM 112 cells with a 5 μM concentration of the HCMV replication inhibitor ganciclovir resensitized the cells to temozolomide (Fig. 7B). We conclude that HCMV-infected GBM cells are resistant to temozolomide but that sensitivity can be reestablished upon ganciclovir treatment. In total, we conclude that HCMV-infected GBM cells display *in vitro* characteristics of cancer stem cells.

## DISCUSSION

Epidemiology studies have limited power to conclusively demonstrate an association of HCMV with human cancers based on the paradigm established by Epstein-Barr virus (EBV), where the virus clearly drives the tumor phenotype but only a minority of those infected will ever develop a virus-mediated tumor (32). Studies to detect the presence of viral genomes, transcripts, or proteins in GBM specimens either conclude that they are present or absent (18), and therefore, additional work of this type is unlikely to move the field forward. Furthermore, the virus does not overtly transform cells *in vitro*, indicating that any initiating or potentiating effects HCMV may have on GBMs are likely to be different than those of classically defined tumor viruses.

Despite the technical challenges and skepticism, interrogation of potential roles for HCMV in GBMs persists because virally induced tumors can be treated with antiviral drugs, and with such a poor prognosis for GBM patients, any potential new therapy deserves exploration. Furthermore, preliminary clinical work seems to indicate that antiviral therapies provide some measure of efficacy against GBMs. Treatment with valganciclovir extended the survival of GBM patients (14, 15). This drug inhibits productive HCMV infection but also inhibits EBV, herpes simplex virus type 1 and type 2, varicella-zoster virus, and human herpesvirus 6 (33). It is unclear whether the positive effects are due to the inhibition of HCMV replication or inhibition of these other viruses. The success of immunotherapies directed at HCMV antigens in GBM patients (16, 17) seems to indicate that curtailing HCMV infection contributes to a positive outcome. In all these studies, it is unclear if the positive effect is mediated by the inhibition of virus replication in the tumor itself or systemically in cancer patients who are likely partially immunocompromised.

Work presented here demonstrates that HCMV-infected primary GBM cells display multiple stem cell-like properties. Previous studies have shown that glioma cell lines propagated as mouse xenografts show increased self-renewal capacity after HCMV infection (34) and that adherent glioma cells cultured in stem cell media grew as single cells when uninfected but as clumps (described as spheres) when infected with HCMV (35). These studies also determined that HCMV-infected cells express CD133, which some believe is a marker for cancer stem cells (36). Our work extends these studies into physiologically relevant cells and additional phenotypic markers of stem cells (slow growth and drug resistance). At present, we cannot determine whether HCMV reverts more-differentiated cells to a stem cell-like phenotype or simply perpetuates the stem cell characteristics already present in the cells it infects.

The slow-growth property we identify in HCMV-infected GBM cells (Fig. 4) likely explains why viral genomes and antigens seem to hover near the limits of detection in GBM samples (18), why our previous work estimated that as few as 1% of the cells within a tumor could possibly harbor viral genomes (19), and our finding here that viral genomes are swiftly diluted *in vitro* under population culture conditions that inherently select for quickly dividing cells (Fig. 1). The HCMV-induced slow-growth phenotype may also directly explain the observed temozolomide resistance, as rapidly proliferating cells are generally more sensitive to chemotherapies.

Our ganciclovir experiment (Fig. 7B) indicates that inhibiting viral replication resensitizes HCMV-infected GBM cells to the chemotherapeutic drug temozolomide. Interestingly, ganciclovir had no effect on the ability of HCMV-infected GBMs to display enhanced sphere formation in this single-round assay. This result permits speculation regarding how viral functions impact tumor growth and survival. Perhaps viral IE or early proteins (not reduced by ganciclovir inhibition of viral DNA replication) mediate the increased self-renewal (34), while later functions (dependent upon viral DNA replication) mediate temozolomide resistance. As HCMV manipulates the DNA damage response (37), it will be interesting to explore exactly how viral replication confers temozolomide resistance.

It is unlikely that experimental approaches will ever definitively prove that HCMV infection is a root cause of GBM. However, our study here allows us to build a scientifically sound and testable model based on experimental data for how HCMV could be contributing to GBMs. We propose that HCMV infection plays no role in GBM initiation but, upon infecting the preexisting tumor, enhances or induces stem cell-like properties in the cells it infects, making them refractory to, and promoting recurrence after, chemotherapeutic treatment. Such a model permits the virus to contribute in a positive way to tumor survival while infecting only a vanishingly small number of cells within the tumor. It also indicates how antiviral therapies in conjunction with current standard of care approaches can prolong survival but likely not cure patients, as uninfected GBM stem cells would resist antiviral therapy. Our work establishes a rationale for future *in vitro* studies examining how HCMV manipulates stem cell-specific pathways and future clinical studies exploring antiviral treatments as adjunct therapies for GBM patients.

## MATERIALS AND METHODS

### GBM acquisition, culture, and infection.

GBM specimens were collected in the operating room under a protocol approved by the University of Wisconsin—Madison’s institutional review board. Portions of the GBM specimens were snap-frozen, and the rest of the tissue was subjected to *ex vivo* culture. Single-cell suspensions were generated as described previously (38). For monolayer culture, patient-derived GBM cells were maintained in Dulbecco’s modified Eagle’s medium (DMEM) supplemented with 10% fetal bovine serum (FBS), 1% penicillin-streptomycin, and 4 mM l-glutamine. For GBM sphere culture, patient-derived GBM cells were maintained in DMEM/F12 1:1 medium supplemented with 2% vitamin B_27_ minus vitamin A, 1% penicillin-streptomycin, 4 mM l-glutamine, 5 μg/ml of heparin sodium, 20 ng/ml of epidermal growth factor (EGF), and 10 ng/ml of basic fibroblast growth factor (bFGF). All the cells were grown in a humidified incubator at 37°C with 5% CO_2_. DMEM/F12 medium, vitamin B_27_, l-glutamine, antibiotics, and FBS were obtained from Thermo Fisher Scientific, Waltham, MA. EGF and bFGF were purchased from PeproTech, Rocky Hill, NJ. DMEM and heparin sodium were purchased from Sigma-Aldrich, St. Louis, MO. For infection, GBM spheres were dissociated with trypsin-EDTA (Thermo Fisher) and resuspended at 1,000 cells/μl in culture media without EGF and bFGF. A total of 5 × 10^5^ to 10^6^ cells were infected with HCMV strain TB40/E labeled with mCherry (TB40/E-mCherry) (39) in a 37°C water bath for 1 h with frequent trituration. Infected cells were then cultured in media with growth factors at 10 cells/μl. At day 7, GBM spheres were dissociated and analyzed with BD LSR II system (BD Biosciences, San Jose, CA) for their mCherry intensity. Dead cells were excluded by LIVE/DEAD violet or LIVE/DEAD near-IR (Thermo Fisher).

### PCRs.

Total DNA and RNA were extracted from GBM tissues and cell cultures with the Allprep DNA/RNA minikit (Qiagen, Germantown, MD). HCMV DNA amplification utilized 100 ng or 250 ng of DNA, the picomaxx master mix (Agilent Technologies, Santa Clara, CA) and 3% dimethyl sulfoxide (DMSO) in a 25-μl volume. The PCR protocol was 95°C for 2 min, followed by 14 touchdown cycles (1 cycle consisting of 95°C for 30 s, 65°C [decreasing by 0.5°C every cycle] for 30 s, and 72°C for 1 min) and then 25 amplification cycles (1 cycle consisting of 95°C for 30 s, 58°C for 30 s, and 72°C for 1 min). Five microliters of product was used as the template for another identical round of PCR. Products were visualized on 1.8% agarose gels. HCMV RNA amplification utilized the removal of DNA contamination by on-column DNase digestion (Qiagen), 100 ng of RNA, and the Promega Access reverse transcription-PCR (RT-PCR) system according to the manufacturer’s protocol. The RT-PCR protocol was 45°C for 45 min, 94°C for 2 min, followed by 35 amplification cycles (1 cycle consisting of 94°C for 30 s, 58°C for 30 s, and 68°C for 30 s). Products were visualized on 1.8% agarose gels. All primer sequences have been previously published (19, 20).

### Plaque assay.

HCMV-infected GBM stem cells were plated at 10 cells/μl in a total volume of 5 ml. Supernatant was collected at 48, 96, and 144 h postinfection (hpi) in three biological replicate experiments. One milliliter of the supernatant was used to infect primary human fibroblasts grown in six-well plates as previously described (40). The next day, the medium was removed, and an agarose overlay was added as previously described (40). After 14 days, plaques were stained with 0.03% methylene blue and counted. For infected GBM 112 cells, released virions from 5 ml of supernatant were concentrated by centrifugation through 20% sorbitol, resuspended in 1 ml DMEM, and then used as described above. To differentiate between cell-free and cell-associated virus, cultures infected as described above were harvested at 144 hpi. The titers of virus in supernatants were determined directly. Cell pellets were resuspended in media, sonicated, and pelleted to remove debris, and the titers of virus in the cell pellets were determined.

### Cell growth assays.

Growth curves were generated under normal culture conditions by counting live cells with a hemocytometer (dead cells were excluded by trypan blue staining). The CFSE assay was performed using the CellTrace cell proliferation kit (Thermo Fisher) according to the manufacturer’s instructions. Briefly, one million cells in 1 ml were incubated with 5 μM CFSE in phosphate-buffered saline (PBS) for 20 min in the dark. Media with growth factors (4 ml) was added to stop the reaction. Cells were centrifuged, resuspended, and grown for 7 days at which time spheres were dissociated and analyzed with BD LSR II system (BD Biosciences). Dead cells were excluded by LIVE/DEAD violet or LIVE/DEAD near-IR (Thermo Fisher). For the sphere formation assay, 1,000 cells at 10 cells/μl were plated in the wells of a 96-well plate. Spheres (clusters with more than 3 cells) were counted on day 7. For the chemoresistance assay, cells were cultured under reduced growth factor conditions (10 ng of EGF and 5 ng of bFGF). Temozolomide and/or ganciclovir (Sigma-Aldrich) was added at 48 hpi. Control groups were treated with the same concentration of DMSO. The numbers of spheres in each well were counted at day 7.

### Data analysis.

Flow cytometry data were analyzed by FlowJo software version 10 (FlowJo LLC, Ashland, OR) All statistical analyses were calculated using GraphPad Prism 7 software (GraphPad Software, Inc., La Jolla, CA). All data are presented as means ± standard errors of the means. *P* values were calculated using Student’s *t* test with a two-tailed distribution. All experiments were performed at least three times except where indicated. Original data will be made available upon request.
